# *acr-23* Encodes a Monepantel-Sensitive Channel in *Caenorhabditis elegans*

**DOI:** 10.1371/journal.ppat.1003524

**Published:** 2013-08-08

**Authors:** Lucien Rufener, Nicola Bedoni, Roland Baur, Samantha Rey, Dominique A. Glauser, Jacques Bouvier, Robin Beech, Erwin Sigel, Alessandro Puoti

**Affiliations:** 1 Novartis Centre de Recherche Santé Animale, St. Aubin, Switzerland; 2 Institute of Parasitology, Macdonald College, McGill University, Ste Anne de Bellevue, Quebec, Canada; 3 Department of Biology, University of Fribourg, Fribourg, Switzerland; 4 Institute for Biochemistry and Molecular Medicine, University of Bern, Bern, Switzerland; Rush University Medical Center, United States of America

## Abstract

Monepantel is a member of the recently identified class of anthelmintics known as the amino-acetonitrile derivatives (AADs). Monepantel controls all major gastro-intestinal nematodes in sheep including those that are resistant to the classical anthelmintics. Previous studies have shown that the *Caenorhabditis elegans acr-23* and the *Haemonchus contortus Hco-mptl-1* genes may be prominent targets of monepantel. With this discovery it became possible to investigate the mode of action of monepantel in nematodes at the molecular level. In the present study, we show that a *C. elegans* mutant *acr-23* strain is fully rescued by expressing the wild-type *acr-23* gene. Moreover, we present a new mutant allele, and characterize *acr-23* alleles genetically. We also show that *acr-23* is expressed in body wall muscle cells, and provide therefore a possible explanation for the paralysis caused by monepantel. Furthermore, genetic evidence suggests that the chaperone RIC-3 is required for expression of full monepantel resistance. Finally, we present reconstitution of the *C. elegans* ACR-23 receptor in *Xenopus laevis* oocytes and provide direct evidence of its modulation by monepantel. Conversely, co-injection of the chaperone RIC-3 had no impact for channel reconstitution in *X. laevis* oocytes. These results reinforce the involvement of the ACR-23 family in the mode of action of monepantel and advance our understanding of this new class of anthelmintics.

## Author Summary

Worldwide, sheep and cattle farming are endangered by anthelmintic-resistant gastro-intestinal nematodes. Monepantel, a member of the recently identified class of anthelmintics known as the amino-acetonitrile derivatives (AADs) exhibits excellent efficacy against various species of livestock-pathogenic nematodes and, more importantly, overcomes existing resistances to the currently available anthelmintics. Previous studies conducted with the non-parasitic model nematode *Caenorhabditis elegans* have found that *acr-23* mutants are fully resistant to monepantel. In this study, we confirm the role of *acr-23* as a primary target of monepantel by 1) restoring monepantel sensitivity in the presence of extrachromosomal copies of wild-type *acr-23*, 2) finding that ACR-23 is mainly expressed in muscle cells, and 3) showing that *acr-23* forms a monepantel-sensitive channel in *Xenopus* oocytes.

## Introduction

Parasitic infections by nematodes represent a serious threat to the health of humans, companion animals and livestock. Three classes of broad-spectrum anthelmintics have been available over the past three to four decades to control these parasites. Unfortunately some of these anthelmintics have, over time, decreased in effectiveness with the appearance of resistant parasite populations [1]. As a result, the development of compounds from new drug classes that are able to control such resistant strains became essential. The recently identified class of AAD anthelmintics offers an attractive solution, either used alone or alternating with older anthelmintic classes. Importantly, the mode of action of the AADs is different from that of other commercially available anthelmintics [2], [3], [4].

To investigate the molecular mode of action of AAD compounds, a genetic screen has been performed in the non-parasitic nematode *Caenorhabditis elegans*, which is amenable to genetic analyses [2]. This screen led to the identification of 44 mutant alleles. Among these, 27 alleles fell into a single class of complementation and were found to disrupt the *acr-23* gene. This suggests that *acr-23* represents a prominent target for AAD molecules [2] including monepantel. Most mutant *acr-23* strains show complete resistance to monepantel when cultured under standard conditions in the presence of micromolar doses of monepantel. Furthermore, the search for parasitic nematodes resistant to monepantel has led to the identification of *Hco-mptl-1*, which is disrupted in monepantel-resistant strains of the parasitic nematode *Haemonchus contortus* [2], [4]. This screen was carried out in natural hosts of this roundworm and confirms that a subunit from the DEG-3 subfamily of receptors represents a major target for monepantel [4]. With the discovery of *acr-23*, it was possible to investigate the mode of action of AADs in nematodes. To date no molecular analysis has been carried out. In particular, it is not known if *acr-23* is solely responsible for the resistance to the AADs and how the ACR-23 protein responds to monepantel.

To answer these questions, we initiated the molecular characterization of *acr-23* in *C. elegans*. We have found that a mutant *acr-23* strain is fully rescued by expressing the wild-type *acr-23* gene. Moreover, the tissues that are targeted by monepantel have been identified through the expression of a fusion protein between ACR-23 and the green fluorescent protein (GFP). In addition, we have tested *C. elegans* mutants of several other subunits closely related to *acr-23* for their sensitivity to monepantel and their genetic interaction with *ric-3*. We also checked for the potential of the *H. contortus Hco-mptl-1* gene to restore monepantel sensitivity in *C. elegans acr-23* mutants. Finally, we present functional expression of the ACR-23 receptor in *Xenopus* oocytes and direct evidence of its modulation by monepantel. Our work represents the first step towards the molecular characterization of the *acr-23* gene and its involvement in the monepantel sensitivity phenotype. This work enhances our understanding on how to control drug-resistant nematodes and additionally supports the development of genetic markers for the early detection of resistant genotypes in the field.

## Materials and Methods

### Strains, transgenics, and behavioural assay

*C. elegans* strains were grown at 20°C based on a previously described general *C. elegans* procedure [5], except where specified. Worms were fed with OP50 *E. coli*. N2 was used as a reference wild-type *C. elegans* strain. Monepantel-resistant mutants were described previously [2] except *acr-23(tm2804)V* and *acr-23(cb110)V*. Transgenic strains AP162 [*acr-23(cb27)V; puIs4*], AP176 [*ric-3(md158)IV; puIs3*] and AP215 [*acr-23(cb27)V; puIs4; puEx33*] were generated by microinjection. *puIs3* and *puIs4* are integrated arrays containing *acr-23(ORF)::gfp::unc-54* 3′UTR and pRF4. *puEx33* is an extrachromosomal array containing *Pmyo-3::mCherry::unc-54 3′UTR*. Other alleles used in this study were *acr-5(ok180)III, acr-23(ok2804)V, des-2(u695)V, deg-3(u662)V*, *acr-18(ok1285)V* and *acr-23(cb92)V*. Alleles *cb103* and *cb98* have not been assigned to *acr-23*, since no mutations were found in the *acr-23* ORF [2]. *ric-3(md158)* is a 5-nucleotide deletion that disrupts the reading frame at the beginning of the second exon. It is very likely to correspond to a molecular null allele [6]. *nT1g* is a semi-dominant marker for chromosomes *IV* and *V*. It is lethal in the homozygous state and confers bright green fluorescence in the pharynx of heterozygous animals. *nT1g* contains a wild-type copy of *acr-23*. Deficiency *eDf43* is a deletion between positions −11.81 and −8.10 on chromosome *V. eDf43* homozygotes reach adulthood but are sterile and uncoordinated.

For the behavioral assay, worms that had been starved for 5 to 6 hours were transferred on fresh NGM-plates and left for 8 to 10 minutes prior to the locomotion measurement. Movies were recorded with a 640×480 pixel USB camera (The imaging Source, Germany) at 7.5 frames per second. Worms were illuminated obliquely with red light-emitting diodes (LED) on a dark background as previously reported [7]. Temperature was held constant at 20°C with a thermoelectric Peltier system coupled to a PID controller (Macshane Inc., Medina, OH). Temperature at the top of the medium was measured with a thermistor probe and fluctuated by less than 0.1°C during each movie. Movies were analyzed with the Multi-Worm Tracker (MWT) software [8]. The position of the center of mass of each object (worm) was smoothed with a three-frame moving average. For each animal, instant speed was calculated for each frame pair. Reversal and steering events were flagged by comparing the angle made by three successive positions of the object (angle range: −180° to 180°). Angles with absolute values over 150° were flagged as reversals. Angles between 30° and 120° were flagged as steering events.

Growth kinetics was performed at 20°C by looking at germline and vulval development, and by scoring the number of laid eggs and hatched progeny. We scored the development of embryos that were either transferred to, or laid directly on plates containing 0, 20 or 60 µM monepantel. To compare growth of *ric-3; acr-23* mutants in the presence or absence of monepantel, we counted progeny of four fertile young adult hermaphrodites after 3, 6 and 9 days at 20°C. In subsequent experiments the scoring was simplified by assigning nine subjective growth levels that take into account the volume of worms and the status of the bacterial lawn after 9 days of culture: Level 1 corresponds to scarce coverage of worms, with an even lawn of bacteria. At Level 2, there is a moderate coverage of worms and patches of bacterial lawn while at Level 3, plates are crowded with worms, and only spots of bacteria are left. Level 4 corresponds to very crowded plates with no remaining bacteria. Plates with starved worms that have dug into the agar are assigned to Level 5. Intermediate levels (1.5, 2.5, 3.5 and 4.5) are also assigned for greater discrimination. Aging was scored at 20°C on 80 wild-type or *acr-23(cb27)* fourth-stage (L4) larvae. We scored body movement and analyzed the data using the Log-rank test of OASIS [9]. Broods were analyzed by transferring gravid hermaphrodites on fresh plates, until they exhausted their sperm supply and laid oocytes instead of embryos.

### DNA sequences

For *acr-23(ok2804)*, the sequences flanking the 684 nt deletion are: 5′ taaattttacattttggaatgattcaata 3′ and 5′ ttaaaatctcaccttttgttccaagaac 3′. Flanking sequences for *acr-23(cb110)* are 5′ ggaaactaaccctatgttatactc 3′ and 5′ atctttgtaggtttgtagtttgtagttctt 3′. *acr-23(cb103)* is a G to A transition changing Alanine_321_ into Threonine. Resequencing of alleles *cb98* and *cb78* did not reveal mutations within the *acr-23* ORF (sequencing covered all exons and the ends of introns).

### Constructions and complementation of mutants

The promoter and genomic coding sequence, including introns from *acr-23* (total of 12,581 bp) was amplified by Polymerase Chain Reaction (PCR) using the Expand Long Template PCR System (Roche) with the following primers: *Ce*-prom_A_frw1 (5′ cattgcctggcaccagagataacaa 3′) and *Ce-acr23*FL_rev1 (5′ aagaaaatgattttccgtataccaaatatacc 3′). The *gfp* coding sequence plus the *unc-54* 3′ untranslated region (UTR) was amplified by PCR from vector pPD95.75 with the following primers: *Cel-acr-23*FL_*gfp*_frw1 (5′ gaattaatttatatggctggtatatttggtatacggaaaatcattttcttagcttgcatgcctgcaggtcgact 3′) and *unc-54* 3′UTR_r1 (5′ aagggcccgtacggccgactagtagg 3′). The *Cel-acr-23*FL_*gfp*_frw1 primer was designed to have a 50-nucleotide overhang to the *Cel-acr-23* gene. Genomic DNA fragments coding for *acr-23* (10 ng/µL) and *gfp* (1.4 ng/µL) were co-injected into adult germlines at equivalent molar ratios. Both fragments recombined in vivo. pRF4 (a dominant allele of *rol-6*) was used as an injection marker at 50 ng/µL [10]. Rescue was also successful with an *acr-23* genomic fragment alone (22 ng/µL). The presence of transgenes and the fusion of homologous products were assessed by PCR. The mutations rescued were *acr-23(cb27)*, *acr-23(cb103), acr-23(cb92)*, but also other alleles such as *cb98* and *cb78*.

The promoter region, the signal peptide and the first four amino acids from *Cel-acr-23* (4841 bp) were amplified by PCR from genomic DNA. The same procedure was used to amplify the *Cel-acr-23* 3′UTR (263 bp). The coding sequence of *Hco-mptl-1* (1642 bp) was amplified by PCR from a cDNA plasmid clone [4] using primers designed to have overlap with the *acr-23* gene including its promoter. All three PCR products were assembled using a fusion-PCR approach. The final PCR fragment was gel purified (NucleoSpin kit, Macherey Nagel) and cloned into a pCRII-TOPO (Invitrogen). Plasmid DNA was purified using the QIAprep Spin Miniprep kit (Qiagen) and sequenced using the standard primers M13 forward and reverse as well as internal primers.

Expression of *acr-23::gfp* was analyzed by epifluorescence and confocal microscopy using several independent transgenic lines. The pictures in the results section come from animals that have integrated the transgene and co-express an extrachromosomal array carrying the *Pmyo-3::mCherry::unc-54* construct (pCFJ104 from addgene).

### Cloning of *acr-23* and *ric-3* cDNA

RNA extraction, cDNA synthesis and PCR amplification were performed as described [4]. In brief, total RNA was extracted from a pool of approximately 50 nematodes, and 1 µg of total RNA (DNase-treated) was reverse-transcribed to cDNA using a (dT)30 primer and SuperScript III Reverse Transcriptase (Invitrogen, Carlsbad, CA). Primer sequences are available on request. Plasmid DNA was purified and at least three clones of each construct were sequenced and compared to the sequences published on Wormbase (www.wormbase.org). The same procedure was used to obtain the *acr-23* sequences with an early (pAP326) or late (pAP323) stop in frame (at position 326 and 1191, respectively) and a D112N substitution (*acr-23(cb101)*) from *C. elegans* mutants. The selected inserts were subcloned into a pT7-TS transcription vector (that introduces *X. laevis* β-globin untranslated DNA to the 5′ and 3′ end of the gene) via the restriction sites inserted in the primers. Plasmid DNA was purified with an EndoFree Plasmid Purification kit (Qiagen).

### Expression of ACR-23 proteins in *Xenopus laevis* oocytes

Capped cRNAs were synthesized (T7 mMessage mMachine kit, Ambion, Austin, TX) from the linearized vectors containing the different subunits. Isolation of oocytes, culturing of the oocytes, and defolliculation was performed as described by Sigel [11], [12]. Oocytes were micro-injected with 50 nl of cRNA solution (0.2 or 0.5 ng/nL) and then stored at 18°C in sterile filtered Barth's modified medium containing NaCl (88 mM), KCl (1 mM), NaHCO_3_ (2.4 mM), HEPES (10 mM, pH 7.5), MgSO_4_ (0.82 mM), Ca(NO_3_)_2_ (0.34 mM), CaCl_2_ (0.41 mM), and penicillin/streptomycin (100 U/mL). Recordings were routinely made 1–3 days post-cRNA injection.

### Two-electrode voltage-clamp measurements

The measurements were performed at the Institute of Parasitology, McGill University (Montréal, Canada) and at the Institute for Biochemistry and Molecular Medicine (IBMM, Bern, Switzerland). Unless indicated otherwise, measurements were done in ND96 medium containing 90 mM NaCl, 1 mM MgCl_2_, 1 mM KCl, 1 mM CaCl_2_, and 5 mM HEPES, pH 7.4, at a holding potential of −80 mV. Glass electrodes containing Ag|AgCl wire were filled with 3 M KCl and had a resistance between 0.5 and 5 MΩ. Currents were measured using a GeneClamp 500B voltage clamp (Axon Instrument) and with a custom-made two-electrode voltage clamp amplifier in combination with an XY recorder (90% response time, 0.1 s) or the current was digitized at 100 Hz using a MacLab/200 (ADInstruments Ltd., Chalgrove, Oxfordshire, UK) at McGill and IBMM, respectively. The amplifier was characterized by a linear response up to 20 µA. Ligands such as acetylcholine, choline-chloride and nicotine (Sigma) were dissolved in ND96 and applied in the absence or presence of modulatory compounds for 20 s. The modulatory compounds were prepared as a 10 mM stock solution in dimethyl sulfoxide (DMSO) and were dissolved in external solution resulting in a maximal final DMSO concentration of 0.1%. Agonists and modulatory compounds were washed over the oocytes using a RC-3Z recording chamber (Warner Instrument Inc.) at McGill. At IBMM, the perfusion solution (6 ml/min) was applied through a glass capillary with an inner diameter of 1.35 mm, the mouth of which was placed approximately 0.4 mm from the surface of the oocyte, enabling 70% solution change in 0.5 s [13]. To avoid contamination, the perfusion system was cleaned between experiments by washing with 100% DMSO after application of the AAD derivatives. Data was obtained and analyzed using Clampex software (Molecular Devices).

## Results

### *acr-23* loss-of-function alleles confer resistance to monepantel

In an attempt to further investigate the implication of the DEG-3 subfamily proteins in their sensitivity to monepantel [2], [4] we characterized monepantel-resistant *C. elegans* isolates that were identified through a large scale genetic screen [2]. Out of 44 alleles recovered, 27 corresponded to mutations in the nicotinic acetylcholine receptor (nAChR) gene *acr-23* [2]. Among these, *acr-23(cb110)* was described as a very strong allele although it was merely a change from Alanine to Valine in the linker domain between transmembrane domains 3 and 4 [2]. However, when we resequenced this allele, we did not find the predicted mutation. Instead, we found a 592-nucleotide deletion that disrupts the *acr-23* open reading frame, thus explaining the strong resistance of the allele *acr-23(cb110)*. Similarly, we found that allele *acr-23(cb103)*, which confers high resistance to monepantel corresponds to a missense mutation in the linker domain of ACR-23. Finally, a new allele of *acr-23* was found by the *C. elegans* knockout consortium, independently of resistance to monepantel. This allele, *acr-23(ok2804)* carries a 684-nucleotide deletion that disrupts the open reading frame before the first transmembrane domain and confers strong resistance to monepantel. This finding represents an additional and independent line of evidence that ACR-23 is a target of monepantel. The sequences of the various alleles are given in the methods section.

**Figure 1:**
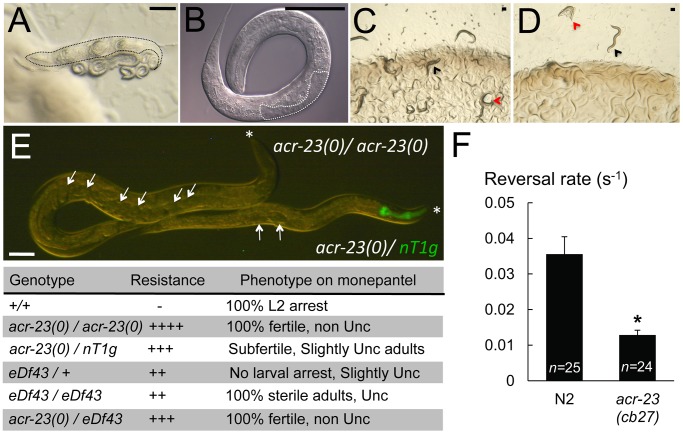
Effects of monepantel on *C. elegans* and rescue of mutant *acr-23*. A) Adult N2 hermaphrodite with hatched embryos on 60 µM monepantel. L1 and L2 larvae are paralyzed and coiled (carcass of mother is outlined). B) Higher resolution of an arrested L2 larva. The germline is outlined. C) *acr-23*(*cb27*) animals carrying an extrachromosomal array containing wild-type *acr-23* grown on standard NGM. Transgenic animals have a rolling phenotype (red arrowhead; non transgenic worms are denoted by a black arrowhead in C and D). D) In presence of monepantel, only non-rolling siblings are mobile and fertile. Transgenic animals are immobilized and dying or dead. Panels C) and D) show plates with the progeny of one single rolling parent incubated for 6 days at 20°C. E) Phenotypes of homozygotic and heterozygotic *acr-23(cb27).* *eDf43* is a deficiency that spans *acr-23* and neighboring genes. Depicted adult worms are one heterozygote, with dominant pharyngeal GFP expression (*nT1g*), and one non-glowing *acr-23(cb27)* homozygote, both grown on 20 µM monepantel. White arrows indicate all embryos present in the uterus. Similar results were obtained with *acr-23(ok2804)/nT1g* heterozygotes (not shown). Asterisks indicate the head. Bar, 50 µm. F) *acr-23* mutants make fewer reversals. The rate of reversal events, where worms shift from forward to backward locomotion or vice versa, was measured in starved worms crawling on food-free NGM plates at 20°C. *n* is the number of movies analyzed, each tracking 15 to 60 individuals over at least 1 minute. *, P-value = 6.4E-5 versus N2 by Student's T-test (two-tailed). Results are expressed as mean with s.e.m as error bars.

As already shown for the benzimidazoles and the gene *ben-1* [14], one would predict that complementing mutant alleles with wild-type copies of *acr-23* would restore sensitivity to monepantel. We therefore transformed monepantel-resistant *acr-23* mutants with wild-type copies of *acr-23*. Transgenic animals also expressed the *rol-6(su1006)* marker DNA that causes a rolling phenotype. Since the transgene was not integrated, we could compare resistance to monepantel between rolling progeny and their non-rolling siblings. The rescue was complete as transgenic adults behaved like wild-type, in that they became paralyzed after 1–2 hours upon exposure to the drug. When touched with a platinum wire, paralyzed animals are stiff suggesting that the paralysis caused by monepantel is spastic, rather than flaccid, as observed for dead worms. Due to paralysis of the parent hermaphrodite, embryos were not laid and the progeny hatched inside the mother. Progeny bearing the wild-type *acr-23* transgene were paralyzed as well (Figure 1A). Such paralyzed animals arrested development at larval stage 2 (L2), as judged by germline development (Figure 1B). When put on normal growth medium, transgenic strains produced 30–50% of rolling siblings, depending on the line. The extrachromosomal array containing *rol-6* and wild-type *acr-23* is therefore transmitted to 30–50% of the progeny. In the presence of monepantel, only non-rolling animals developed into fertile and mobile adults, while 100% of rolling transgenic siblings were paralyzed and arrested their development (Figure 1C,D).

In the parasite *H. contortus*, the gene with the highest similarity to *acr-23* is *Hco-mptl-1*. Several independent mutations were found in the *Hco-mptl-1* gene from *H. contortus* mutants with reduced sensitivity for monepantel, implicating MPTL-1 as a likely target for AAD action against *H. contortus*. [4]. We tried to complement monepantel resistance of *acr-23(cb27)* mutants with a wild-type *Hco-mptl-1* cDNA driven by the *C. elegans acr-23* promoter. Although we obtained several stable transgenic lines, we never observed complementation of *acr-23* with *H. contortus Hco-mptl-1* (data not shown). One possible explanation is the high degree of divergence between MPTL-1 and ACR-23 amino acid sequences (48.5% identity and 66.8% similarity; [4]). Another reason could be the absence of introns in our transgene. There is evidence of gene regulatory signals in introns and evidence that gene expression and tissue specificity can be increased by including introns [15], [16]. We have used cDNA and not gDNA in our transgene because the *Hco-mptl-1* genomic sequence was too long (>20 kbp) making its amplification difficult. However the implication of *mptl-1* in rendering parasitic nematodes sensitive to monepantel is valid because we have been able to reconstitute a monepantel sensitive MPTL-1 channel in *Xenopus* oocytes (L. Rufener, personal communication).

### *acr-23* alleles are haplo-insufficient

While *acr-23(cb27)* homozygotes are fully resistant to monepantel [2], we found that *acr-23(cb27)/nT1g* or *acr-23(ok2804)/nT1g* heterozygotes are partially affected by the drug. In the presence of 20 µM monepantel, heterozygotes are slightly impaired in their movement while homozygotic mutants move normally. In addition, in the presence of the drug, *acr-23(cb27)/nT1g* heterozygotes produced significantly fewer embyros than their homozygotic siblings: *acr-23(cb27)* homozygotes made 252±23 embryos (n = 8 broods counted) and 237±39 (n = 6) on 2 µM and 20 µM monepantel, respectively. Conversely, *acr-23(cb27)/nT1g* heterozygotes laid only 161±36 (n = 7) and 129±25 (n = 8) eggs under the same conditions. When grown on monepantel, *acr-23(cb27)/nT1g* or *acr-23(ok2804)/nT1g* heterozygotes are also thinner and contain fewer embryos in utero than homozygotes (Figure 1E). Such a semi-dominant effect could be indicative for gain-of-function alleles. However, the molecular lesions of most *acr-23* alleles indicate that they are loss-of-function mutations. We tested this hypothesis with the *eDf43* deficiency, which covers the *acr-23* locus and several neighboring genes. We found that *eDf43+* heterozygotes are resistant to monepantel in that no paralysis or L2 larval arrest were observed (Figure 1E). We also tested *eDf43* homozygotes. Such animals are viable, in spite of the large deletion which causes uncoordinated movement and sterility. In presence of monepantel, *eDf43* homozygotes originating from a heterozygotic mother developed throughout adulthood (Figure 1E). Therefore resistance to monepantel is likely to be caused by loss of *acr-23* activity. Taken together, our results indicate that heterozygotic animals are resistant to monepantel because *acr-23* alleles are haplo-insufficient. In other words, full sensitivity to monepantel requires 2 functional copies of *acr-23*.

### *acr-23* mutants move faster and reverse less frequently

Although *acr-23* mutants are largely resistant to monepantel, they have no obvious phenotype under standard culture conditions [2]. In fact, we found similar growth rates of both wild-type and *acr-23(cb27)* worms. Both took 70 hours to develop from the deposited embyro to the fertile adult. In presence of 20 µM monepantel, *acr-23(cb27)* grew at the same rate indicating that micromolar concentrations of monepantel do not interfere with the speed of embryonic and larval development. We also tested longevity and did not notice differences between wild-type and *acr-23(cb27*) animals: mean life spans were of 18.42±0.37 days for wild-type and 17.04±0.56 days for *acr-23(cb27)* (P-value = 0.1901; n = 80 worms per strain). We did not observe obvious morphological or locomotion defects in *acr-23* mutants. We therefore decided to explore the behavior in more detail. Using quantitative analysis of locomotion, we found subtle alterations in the mutant. When crawling on food-free medium, *acr-23(cb27)* animals had a slightly increased average speed (0.103 body lengths/second ±0.003 for *acr-23(cb27*) versus 0.085±0.004 for N2; P-value = 1.1E-3 by Student's t-test; n≥24 experiments with at least 15 worms each) and more straight trajectories, concomitant with a decreased steering rate (0.35 s^−1^±0.03 for mutant and 0.54±0.04 for wild-type, P-value = 4.1E-7; n≥24). We also observed a decreased frequency of spontaneous reversals (Figure 1F).

### ACR-23 is expressed in body wall muscles

**Figure 2:**
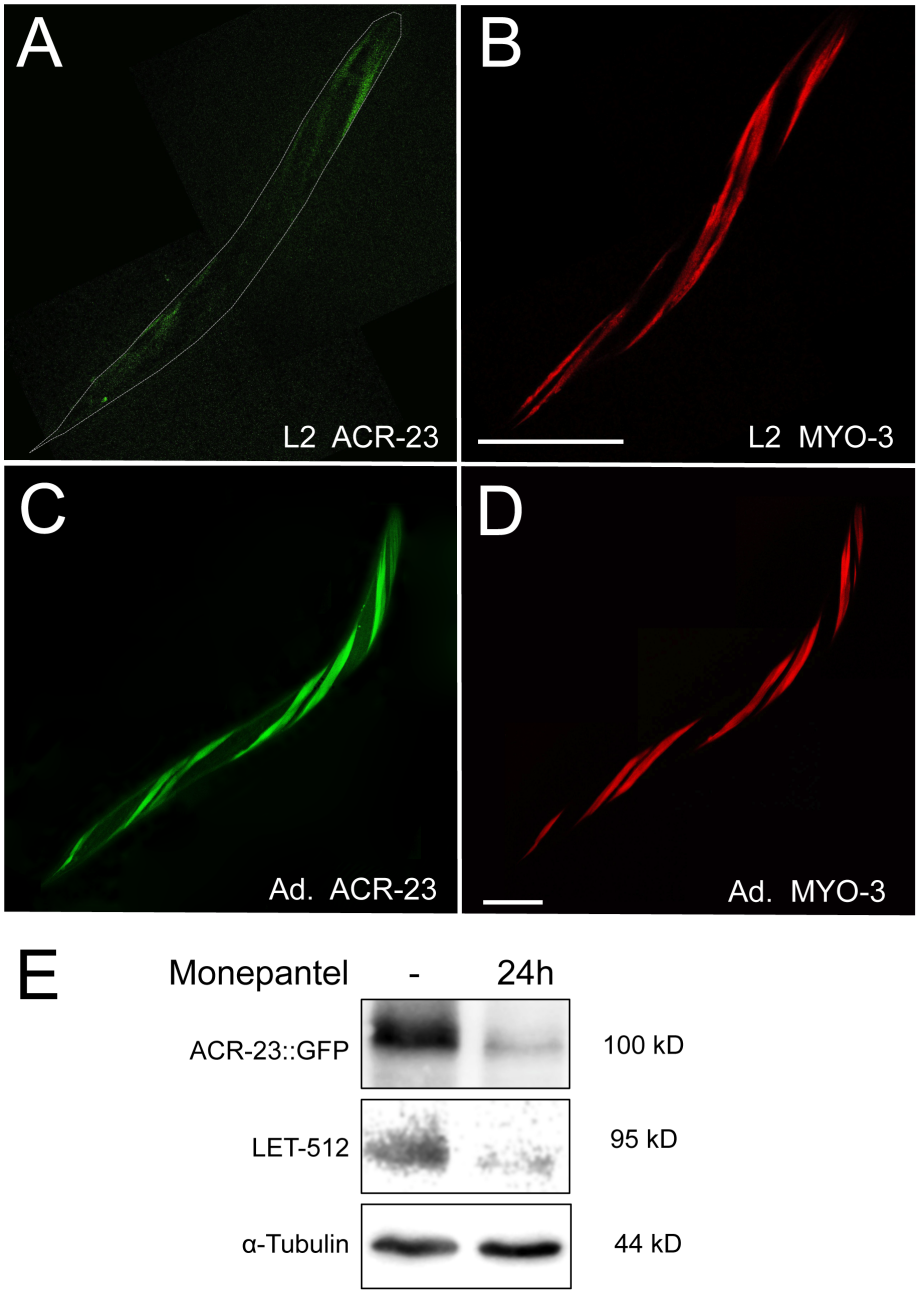
ACR-23 protein during *C.elegans * development. A) L2 larva expressing a *acr-23::gfp* transgene. Twisted morphology is caused by the transformation marker *rol-6*(*su1006)*. B) Same worm expressing *myo-3::mCherry*. C) and D) Expression in the adult. Bar, 50 µm. E) Analysis of ACR-23::GFP expression in response to monepantel after 24 hours of exposure. Loading and general protein expression were tested with α-tubulin and LET-512, respectively.

In order to identify tissues that express ACR-23 and are therefore targeted by monepantel, we generated transgenic *C. elegans* that produce an ACR-23::GFP fusion protein driven by the *acr-23* promoter. The transgene was functional since it rescued mutant *acr-23* animals by restoring full sensitivity to monepantel. By analyzing several independent lines, we found that an integrated transgene expressed mainly in the body wall muscle cells, as well as in a few unidentified head and tail neurons. The fusion protein was visible from larval stage two, where it expressed at low levels. Robust expression was observed from the third larval stage on to adulthood (Figure 2A–D). Co-expression of *myo-3::mcherry* was visible in the same cells, indicating that ACR-23 is expressed in body-wall muscles [17], [18]. No ACR-23::GFP expression was found in embryos and first stage (L1) larvae. Therefore, the onset of ACR-23 expression at larval stage 2 correlates with the observed L2 arrest in response to monepantel. To test whether monepantel regulates ACR-23 expression, we monitored ACR-23::GFP fusion protein levels in the absence and presence of the compound. After 24 hours, worms were paralyzed by the drug. We found that ACR-23::GFP levels were dramatically low after exposure to monepantel (Figure 2E). However, this effect was also observed with PI-kinase LET-512 [19], indicating that monepantel may downregulate general protein synthesis which could also simply be the consequences of dying worms rather than the drug directly acting on gene expression level.

### Functional characterization of the ACR-23 receptor

#### Functional expression in *Xenopus laevis* oocytes

**Figure 3:**
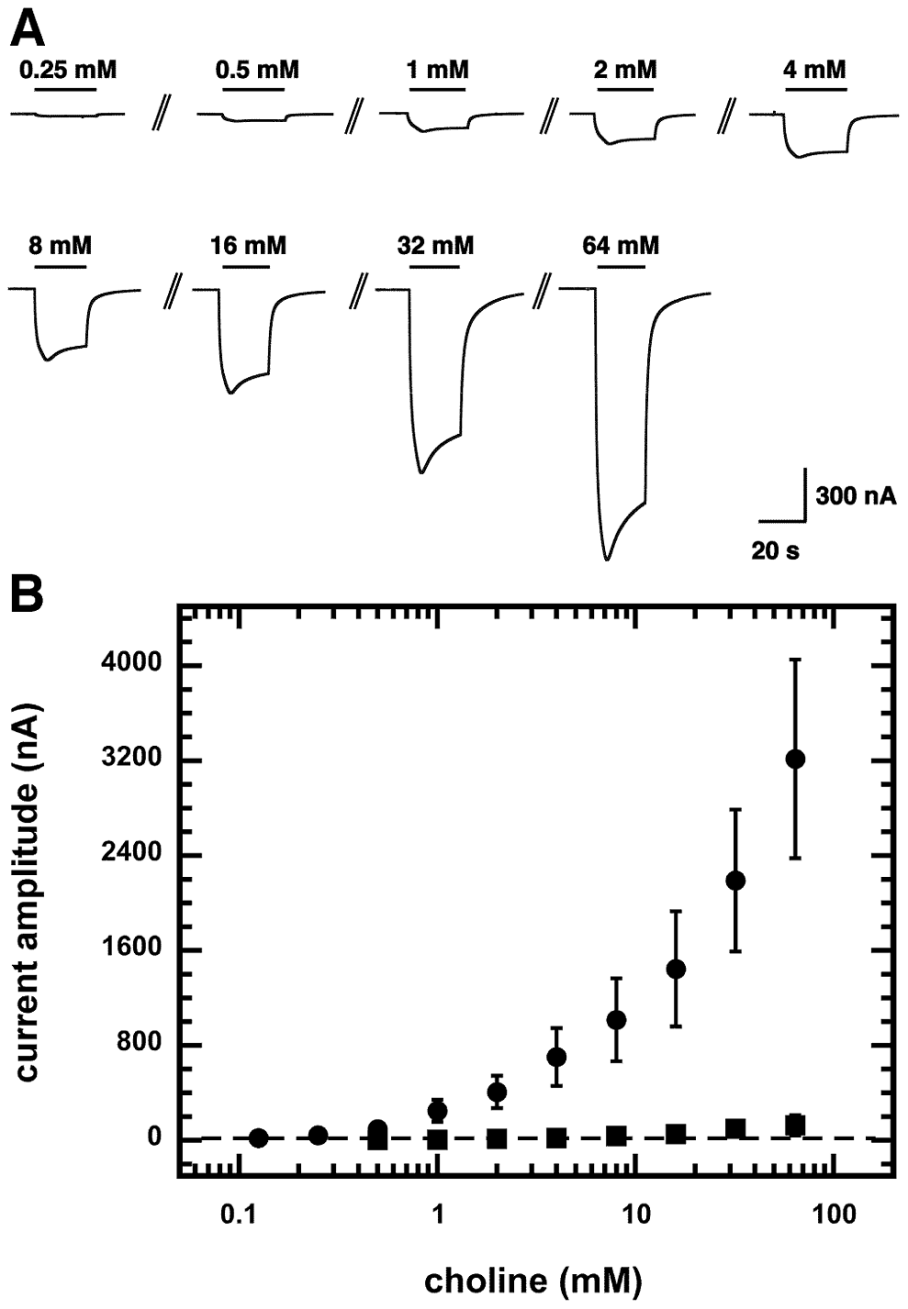
Choline concentration dependence. A) Current traces from a choline concentration response curve obtained from a *Xenopus* oocyte expressing ACR-23 receptors. The bars indicate the time period of choline perfusion. Choline concentrations are indicated above the bars. B) Current amplitude measured in *Xenopus* oocytes expressing a wild-type ACR-23 receptor (filled circles) or a mutant ACR-23 receptor (filled squares) after choline perfusion. The mutant ACR-23 subunit corresponding to allele *acr-23(cb101)* bears a D112N substitution in the extracellular loop. Mean ± SD of experiments carried out with 4–5 oocytes from two batches are shown.

48 hours after injection of *acr-23* cRNAs into *Xenopus* oocytes, currents were elicited by the addition of acetylcholine, choline or nicotine. 30 mM choline elicited peak current amplitudes of 1685±269 nA (n = 9). Currents elicited by choline were characterized by a fast channel opening followed by a slow desensitization. The oocytes rapidly recovered to the baseline currents upon agonist removal. An individual concentration-response curve with choline as the agonist is shown in Figure 3A. No saturation was reached even at higher choline concentrations (Figure 3A and B, filled circles). Injection of mutant *acr-23(cb101)* cRNA produced only weak currents (Figure 3B, filled squares), with peak current amplitudes of 75±100 nA (n = 5) elicited by 64 mM choline. No detectable current could be recorded in oocytes injected with nonsense-mutated *acr-23* cRNAs (pAP326 and pAP323), even at high concentrations of choline (100 mM, data not shown).

#### Choline is the preferred agonist

Choline proved to be a much more potent agonist of the ACR-23 channels than acetylcholine. Acetylcholine (10 mM) elicited only 0.16±0.04% (n = 4) of the current amplitude obtained with 1 mM choline (data not shown). Peak current amplitudes of 653±44 nA (n = 7) were recorded after exposure to 10 mM nicotine (data not shown), which represent 40.4%±2.2% of the currents elicited by 10 mM choline in the same oocytes. Ivermectin was not able to agonize the channel since no recordable current was recorded after the application of the compound at 1 µM (data not shown).

#### Ion selectivity of ACR-23 channels

We previously found that currents mediated by choline activated *H. contortus* DEG-3/DES-2 channels permeated Ca^2+^ ions in addition to monovalent cations [20]. This was detected indirectly by activation of a Ca^2+^-activated chloride channel endogenous to the oocyte, resulting in a shift of the reversal potential towards the chloride reversal potential. It was interesting to see if ACR-23 channels were permeable to Ca^2+^-ions. Instantaneous I–V curves were recorded in standard medium (1 mM CaCl_2_), low calcium medium (standard medium without CaCl_2_, plus 0.1 mM K-EGTA) and high calcium medium (standard medium supplemented with additional 9 mM CaCl_2_) in the presence or absence of 30 mM choline. The reversal potential of the current elicited by choline was 7.3±2.8 mV (n = 4) in standard medium, 7.3±3.1 mV (n = 4) in low calcium medium and 5.0±2.2 mV (n = 4) in high calcium medium. The reversal potential for chloride ions was determined as −26.0±1.0 mV (n = 4), monitoring current through recombinant α1β2γ2 GABA_A_ receptor channels. The fact that the reversal potential does not significantly vary with the conditions indicates that the channel is not permeable to Ca^2+^-ions. The reversal potential in potassium medium, in which all Na^+^ was replaced by K^+^, was measured at 8.3±2.9 mV (n = 4). Presumably, the channel is permeable to both sodium and potassium ions.

#### Effects of monepantel on ACR-23 channels

**Figure 4:**
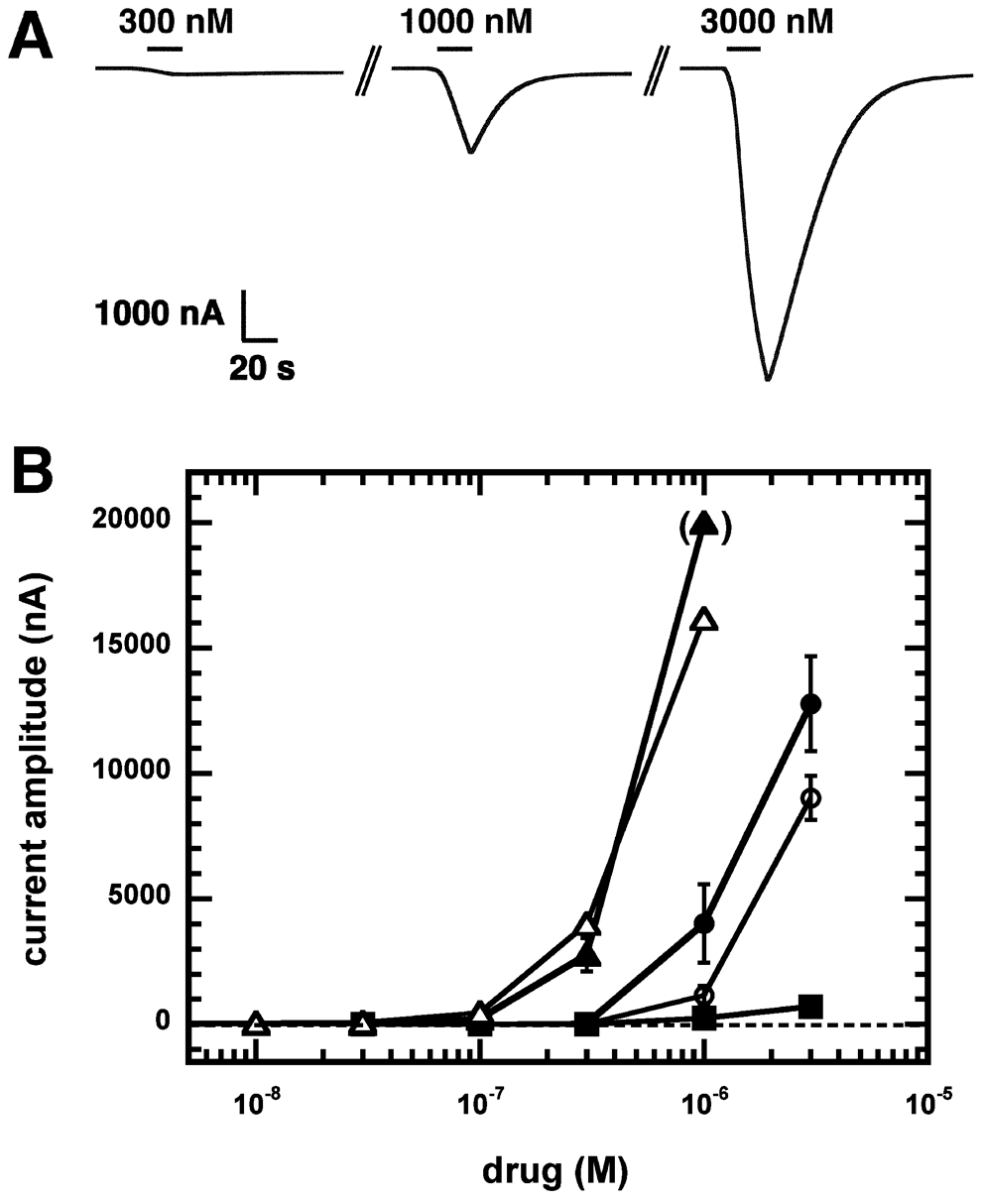
Monepantel is a direct agonist of ACR-23 channel. A) Current traces obtained from a *Xenopus* oocyte expressing ACR-23 upon perfusion with monepantel. The bars indicate the time period of monepantel perfusion. Monepantel concentrations are indicated above the bars. B) Averaged monepantel current amplitudes measured with monepantel (filled circles), monepantel sulfone (open circles) and with monepantel (filled triangles) and monepantel sulfone (open triangles) supplemented with 0.3 mM choline. One point is in brackets as the measured current reached the linear limit of the amplifier (20 µA), preventing a precise measurement. The effect of monepantel on mutant ACR-23(*cb101*) is shown by the filled squares. Mean ± SEM of experiments carried out with 3–6 oocytes from two batches are shown.

To evaluate the effect of monepantel on ACR-23 receptors, the drug was tested as an agonist or as an allosteric modulator of choline. In the absence of choline, monepantel showed a strong agonistic effect on the channel at concentrations higher than 0.3 µM. Figure 4A shows current traces of a concentration response curve of monepantel. After exposure to the compound, the washing time necessary to recover the initial membrane current was much longer than with choline. As monepantel is a hydrophobic compound, it may stick to the membrane of the oocytes making wash-out slower. This, together with the fact that monepantel opens the ACR-23 receptors more slowly than choline, created this typical “V” shape current curves. At concentrations higher than 3 µM, monepantel elicited currents that were too large to be controlled with our amplifier. As no saturation could be reached, it was not possible to measure an EC_50_ value. Nevertheless, when a dose response curve was fitted to the data, the estimated Hill coefficient of individual curves was higher than 2 indicating that several molecules of monepantel must occupy the receptor to open it. The fact that 3 µM monepantel elicited much larger currents than 64 mM choline indicates that choline might be a partial agonist of the channel. Non-injected oocytes failed to respond to monepantel.

When monepantel was co-applied with 0.3 mM choline, the measured currents were larger than with the drug alone and a modulatory effect was evident at 0.1 µM monepantel (Figure 4B, filled triangles and circles, respectively). The monepantel-mediated potentiation of the current was measured at a concentration of 0.3 mM choline, which elicits only a small fraction of the maximal current amplitude. It is interesting to note that the same AAD-modulatory effect could be observed when monepantel was co-applied with 1 mM acetylcholine or nicotine (data not shown). In addition we have tested three different *acr-23* constructions harboring early (pAP326) or late (pAP323) stop codons, or a D112N substitution in the extracellular loop, like in *acr-23(cb101)*. No detectable current could be recorded after injection of the nonsense mutant *acr-23* cRNAs, even when high doses of monepantel (10 µM) were applied. The sensitivity to monepantel of the ACR-23(*cb101*) channel was drastically reduced as 3 µM monepantel generated only 10% of the current measured with the wild-type ACR-23 receptor at the same drug concentration (Figure 4B, filled squares). The Aspartic acid_112_ residue is located in the A-loop of the extracellular domain of ACR-23 [2]. This and several other loops in the extracellular domain of ACR-23 are known to be important for binding agonists [21]. As expected, we measured dramatically reduced currents with the mutant ACR-23(*cb101*) receptor. Co-injection of wild-type *acr-23* cRNA, with or without *ric-3* cRNA, did not significantly affect the modulation of *C. elegans* ACR-23 channels by monepantel (data not shown, n = 3).

We tested the effects of monepantel sulfone on the ACR-23 receptor as this compound is the major metabolite of monepantel in sheep [22]. It forms within hours of animal treatment, and has a longer half-life than monepantel. The sulfone form of monepantel was found to behave similarly to its parent compound, when used alone or in combination with choline (Figure 4B, open circles and triangles, respectively). The optical R-enantiomer of monepantel (AAD-2224), which has no nematocidal effect, was inactive, and failed to elicit current on its own or to potentiate choline responses (data not shown, n = 3).

### The chaperone RIC-3 is required for expression of full monepantel resistance

**Figure 5:**
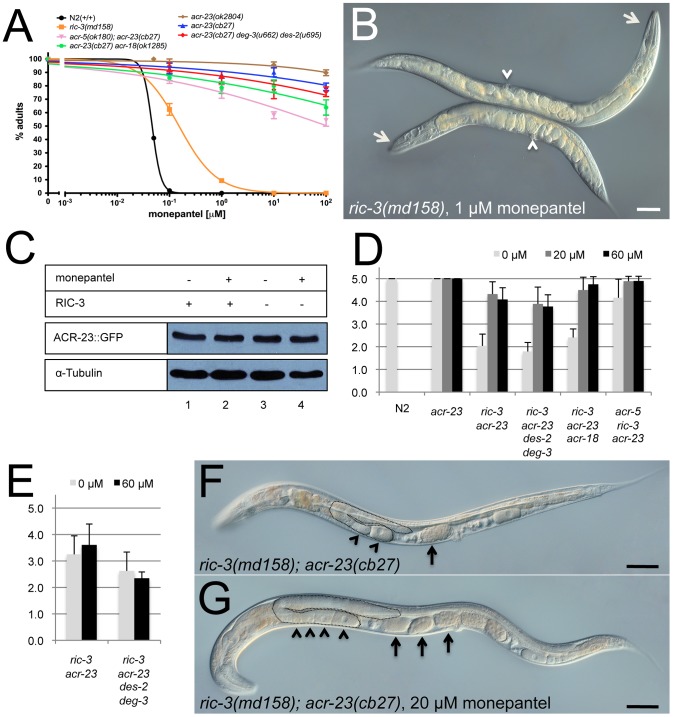
Effect of *ric-3* on the response to monepantel. A) Dose response to monepantel. The average number of progeny reaching adulthood after 3 days is plotted versus the concentration of monepantel. *ric-3(md158)* mutants are partially resistant to low doses of monepantel. B) *ric-3(md158)* adult grown on 1 µM monepantel (bottom) compared to a sibling grown in absence of the drug (top). The reduced length is certainly caused by muscle contraction due to monepantel. White arrows and arrowheads point at the heads and vulvae, respectively. C) ACR-23::GFP protein levels in wild-type (lanes 1,2) and *ric-3* (lanes 3,4) animals after 2 hours of exposure to 10 µM monepantel (lanes 2,4). α-Tubulin was used to verify equal loading. D) Growth of wild-type and mutant strains on monepantel. Light gray (0 µM), dark gray (20 µM) and black (50 µM) bars represent the growth rate after 9 days of culture. In the absence of monepantel, wild-type (N2) worms grow much faster than any of the mutants tested. Genotypes are indicated. Values for growth rates are defined in the methods section (1, poor growth; 5, robust growth). *acr-23* single mutants grew to level 5 in less than 5 days, whereas *ric-3; acr-23* double mutants reached level 2 in absence of monepantel, and level 4 in presence of the drug. E) Growth rates after 9 days on the inactive R-enantiomer of monepantel. F) *ric-3(md158); acr-23(cb27)* adult grown in absence of monepantel. G) Adult *ric-3(md158); acr-23(cb27)* sibling grown on 20 µM monepantel. In both panels (F and G), one germline arm is outlined. Arrowheads and arrows point at oocytes and early embryos, respectively. The outlined germline arm shows robust production of gametes (panel G). Scale bar, 50 µm.

The RIC-3 protein is required for the assembly and trafficking of several nAChR channels and is therefore necessary for their function in *C. elegans* [6]. Under standard conditions, homozygous *ric-3(md158)* mutants grow slowly, are sub-fertile and slightly impaired in their movement. We found that *ric-3* mutants are moderately resistant to monepantel. In fact, after four days of growth in presence of 0.1 µM monepantel, 63% of *ric-3(md158)* mutants developed into fertile adults, while 98% of N2 wild-type worms arrested development as L2 larvae. However, when treated with 10 µM monepantel, 100% of *ric-3* animals arrested development, while *acr-23(cb27)* or *acr-23(ok2804)* mutants were mostly unaffected, since 90% reached adulthood (Figure 5A). Loss-of-function mutations in additional nAChRs proteins such as ACR-18, ACR-5, DES-2 and DEG-3 do not increase the resistance to monepantel (Figure 5A). Similarly, the removal of ACR-5 did not increase the slight resistance of *ric-3(md158)* mutants (data not shown). When treated with low doses of monepantel, *ric-3* mutants, albeit developing into fertile adults, are typically shorter than untreated siblings (Figure 5B). We propose that the shorter size is due to muscular contraction caused by the anthelmintic. Our results suggest that *ric-3* mutants are weakly resistant to monepantel indicating that RIC-3 is required to cause maximal drug effect. We hypothesized that RIC-3 could function in promoting ACR-23 expression. If so, inactivation of *ric-3* might result in improper expression and activity of ACR-23 or, of other proteins that regulate ACR-23 or respond to monepantel. To test this hypothesis, we compared ACR-23::GFP expression in wild-type and *ric-3(md158)* mutant animals, both in the presence and absence of monepantel. Epifluorescence microscopy (data not shown) and western blot analyses did not reveal significant differences of ACR-23 expression (Figure 5C). These findings suggest that RIC-3 does not regulate ACR-23 expression. We also tested a possible synergy between RIC-3 and ACR-23 in the response to monepantel. To do so, we constructed a *ric-3; acr-23* double mutant. Similar to the single *ric-3* mutant, the double mutant grew very slowly, as it took 7 days at 20°C, for an embryo to develop into a fertile adult. As a comparison, wild-type animals take three days. Similarly, *ric-3; acr-23* animals are slightly impaired in their movement, grow slowly, and carry reduced numbers of embryos, indicating that they are not totally healthy [6]. We were however surprised to notice that in the presence of 20 µM monepantel, *ric-3; acr-23* animals grew faster and healthier than in its absence (Figure 5D). In fact, in the presence of the drug, such double mutants had increased mobility and fertility. When we compared the average morphology of treated and untreated *ric-3; acr-23* double mutants, we found that in the absence of monepantel, among 20 randomly picked adults, 11 were clearly sub-fertile, with a small gonad, few and small oocytes and reduced numbers of embryos in the uterus (Figure 5F); eight had an intermediate phenotype and one grew into a fertile adult with a normally shaped germline (Figure 5G). When treated with 60 µM monepantel, *ric-3; acr-23* siblings developed as follows: one was clearly sub-fertile, 13 were intermediate and nine had a normal germline as shown in panel G. Thus monepantel partially rescues the fertility defect caused by the *ric-3* mutation in animals lacking ACR-23. This observation indicates that proteins other than RIC-3 and ACR-23 can mediate the biological effects of monepantel.

To explain increased fertility in the presence of monepantel, we reasoned that if the compound hyper-activated ACR-23 (Figure 4), it might also hyper-activate other nAChR proteins. We also assumed that such nAChRs rely on RIC-3 for robust function [6]. Therefore hyper-activation by monepantel could compensate for reduced nAChR function caused by the loss of RIC-3. As a consequence monepantel would restore more efficient growth of *ric-3* mutants. To verify this and to identify putative secondary targets of monepantel, we tested several homologs of ACR-23: ACR-5, ACR-18 and the combined DES-2/DEG-3 proteins [20], [23]. We therefore compared the growth rate of the corresponding mutants in the presence and absence of monepantel. Loss-of-function mutations in either *des-2 deg-3*, or *acr-18* did not suppress the enhanced fertility on monepantel (Figure 5D). However *acr-5; ric-3; acr-23* triple mutants were less affected by monepantel in that their growth was more robust either in the absence or presence of monepantel (Figure 5D). As a comparison, it should be noted that *acr-23* single mutants grew much more efficiently than any mutant combination containing *ric-3(md158)*. Our findings suggest that neither ACR-18 nor the DES-2 and DEG-3 subunits act as secondary targets of monepantel. However, an *acr-5* mutation partially rescues the slow growth of *ric-3* mutants, but independently of monepantel. The inactive enantiomer of monepantel did not lead to increased fertility of *ric-3; acr-23* and *ric-3; acr-23 des-2 deg-3* mutants indicating that the effect is specific to the enantiomer, which has an anthelmintic activity (Figure 5E).

## Discussion

### Wild-type ACR-23 is targeted by monepantel

The implication of DEG-3 subfamily channels in sensitivity to monepantel is based on mutations found in nematodes that are resistant to AADs [2], [4], [20]. Currently, 29 loss-of-function alleles that confer resistance to monepantel map to the *acr-23* locus. Therefore, a better understanding of AAD action requires a more profound investigation of *acr-23* and the identification of other putative targets of AADs in *C. elegans* and other species. In this report, we provide three lines of evidence that ACR-23 is responsible for the anthelmintic activity of monepantel in *C. elegans*. First, wild-type *acr-23* complements resistance to monepantel. Second, ACR-23 is expressed in body wall muscles, which are paralyzed upon treatment with monepantel. Third, monepantel elicits a current from ACR-23 channels expressed in *Xenopus* oocytes.

The simplest interpretation of the finding that sensitivity to monepantel in *acr-23* loss-of-function mutants can be restored by adding wild-type copies of this gene, is that *acr-23* is a main target of monepantel. This point is strengthened by electrophysiological experiments showing that ACR-23 expressed in *Xenopu*s oocytes reacts to monepantel. However, we also found that alleles (e.g. *cb98* and *cb78*) that do not correspond to coding sequence alterations in the *acr-23* ORF ([2] and this study), were rescued by *acr-23*. We hypothesize that such alleles could affect either promoter or other regulatory regions of *acr-23*. Alternatively, these alleles could correspond to regulatory proteins that control ACR-23 expression or function, and may therefore represent a possible resistance site to monepantel action. Therefore the cloning of the remaining monepantel-resistant alleles represents an important step towards the understanding of the mode of action of AAD anthelmintics.

Direct evidence for the interaction of AADs with a DEG-3 subfamily channel has been reported only once, where the *H. contortus* DEG-3/DES-2 receptor was successfully expressed in *Xenopus* oocytes [20]. It was shown that monepantel sulfone was able to stimulate the choline-activated currents of *H. contortus* DEG-3/DES-2 receptors in a concentration-dependent way, acting as a type II positive allosteric modulator. However, three observations argue against the fact that *H. contortus* DEG-3/DES-2 receptor could be identical to the primary target of the AADs. First, almost all major mutations confering monepantel resistance in *C. elegans* or *H. contortus* have been found in the ACR-23 or MPTL-1 subunits respectively [2], [4]. Second, of the different AADs tested on this channel, only a half were able to stimulate the channel when all of them had been shown to be active *in vitro* [20]. Third, species lacking an ACR-23/MPTL-1 homologue but having a DEG-3/DES-2 channel (e.g. *Pristionchus pacificus* or *Strongyloides ratti*) were shown to be resilient to monepantel treatment [3]. We now show that the presence of wild-type ACR-23 in *C. elegans* is necessary and possibly sufficient for the response to monepantel.

### ACR-23 functions as a monepantel-sensitive ion channel in muscle cells

The effect of monepantel is visible at most developmental stages and results in severe paralysis. When gravid adults were placed on the drug, they became paralyzed as well as their progeny *in utero*. These progeny arrested development at larval stage 2. We found that an ACR-23::GFP fusion protein that rescues *acr-23* mutants is mainly expressed in body wall muscle cells. The expression was visible from larval stage L2 to adulthood. The correlation between ACR-23 expression starting at L2 and the developmental arrest at the same stage corroborates the notion that ACR-23 is a primary target of monepantel. However, we also found that L1 larvae that hatched from eggs transferred on monepantel are severely impaired in their movement, although they do not arrest development and keep developing until L2. We postulate that the sensitivity to monepantel at larval stage 1 might rely on additional proteins that are sensitive to monepantel. As an alternative, the amount of ACR-23 might be too low to be detected early during development. ACR-23 was found in body wall muscles that are arranged into four longitudinal bundles. Body wall muscle cells have been stained with anti-MYO-3 antibodies [18]. *acr-23::gfp* and *myo-3::mCherry* transgenes expressed in the same cells. Although nAChR proteins are expected to be found at neuromuscular junctions, we did not see spots with higher ACR-23 expression. Individual *C. elegans* body wall muscles have both cholinergic and GABAergic inputs, which initiate contraction and relaxation, respectively. A single gene (*unc-49*) encodes the ionotropic GABA receptors present at the *C. elegans* neuromuscular junction (NMJ) [24], [25], [26], [27], [28], [29]. In contrast at least four cholinergic receptors (*unc-38, unc-29, unc-63* and *acr-16*) are found at the NMJ of *C. elegans* [28], [29], [30]. Genetic ablation of those cholinergic receptors has been shown to abolish all cholinergic synaptic currents at the NMJ and severely impair *C. elegans* locomotion [30]. Consequently, we propose that the diffuse distribution of ACR-23 in muscle cells and its apparent absence from the NMJ may correspond to a non-synaptic function of this receptor in body wall muscles. In *Ascaris suum* muscle for instance, Pennington and Martin concluded that extrasynaptic receptors of this parasite contained two types of acetylcholine-activated ion channels, which could serve as possible sites of action for anthelmintic drugs [31]. Although we cannot fully exclude the possibility that other subunits contribute to the native ACR-23-containing receptor, and that the unidentified head and tail neurons could account for the effect of monepantel, we suggest that ACR-23 may function as an extrasynaptic homomeric nicotinic receptor in the *C. elegans* body wall muscles.

### Is choline the natural agonist of ACR-23 channels?

So far, four different receptors of the DEG-3 subfamilly of receptors (DES-2/DEG-3 and ACR-23 from *C. elegans*, and DES-2/DEG-3 and MPTL-1 from *H. contortus*) have been successfully reconstituted in *Xenopus* oocytes ([20], [23], [32]; unpublished data). For all of them, choline always proved to be a much more potent agonist than acetylcholine. But in the case of the ACR-23 receptor, the fact that micromolar concentrations of monepantel elicited much larger currents than millimolar concentrations of choline, and that no current saturation could be obtained, indicates that choline might only be a partial agonist of the channel. To further improve our understanding of the ACR-23 channel properties, it may be of interest to screen a panel of different choline analogues to search for a more potent agonist. As mentioned above, data showing that ACR-23 subunits form a homomeric receptor in vivo are still missing. Therefore, one could argue that a missing subunit may explain the reduced sensitivity towards acetylcholine when expressed in *Xenopus* oocytes. Interestingly, it was shown that choline is interacting with the vertebrate α7 receptors that are expressed in developing muscles prior to synapse formation [33]. Due to this sensitivity to choline [34], the α7 nAChR can be chemically excited, even after the natural neurotransmitter (acetylcholine) has been enzymatically cleaved. As a consequence, chemical neurotransmission involving the α7 nAChR is not limited to short distances as is the case for the other nicotinic receptors. Fischer et al. showed that the α7 nAChR subtype is playing an important role in the rat muscle development for the endplate formation and/or stabilization [33]. It has been reported that choline causes depolarization of muscle membranes [35], [36], and that the choline-evoked depolarization is enhanced after the muscle denervation [33], [37]. Choline also increases the intracellular Ca^2+^ concentration at the muscle endplates [38]. While the in vivo function of the ACR-23 protein remains to be elucidated we may speculate about it based on the present findings. As ACR-23 has a preference for choline and is located extrasynaptically on body wall muscle cells, a possible function could be to increase muscle excitability by slightly depolarizing the cells, thus making a contraction event more likely. By hyper-activating this receptor, monepantel creates a general hyper-contraction of the body wall muscles in nematodes, which are as a consequence, completely paralyzed.

### ACR-23 function in *C. elegans*

nAChRs are known to be necessary for mediating neuronal stimulation and modulation [39]. In nematodes, they have been implicated in various essential functions, like locomotion, egg laying or pharyngeal pumping. ACR-23 belongs to the nematode specific DEG-3 subfamilly of ionotropic nAChRs, which include eight members in *C. elegans* [3], [40]. DEG-3, DES-2 and ACR-5 expression is restricted to neurons [40], while the expression of the five other members has not been reported. Thus, to our knowledge, ACR-23 is the first member of the DEG-3 subfamily known to be expressed in muscles. The lack of an obvious mutant phenotype in the absence of monepantel suggests that ACR-23 is partially redundant with other nAChRs. However, using computer-assisted locomotion analyses, we identified subtle alterations in behavior, with decreased rates of spontaneous reversal and steering, and slightly increased average speed. Since ACR-23 is expressed both in body wall muscles and in yet unidentified neurons, more work will be required to determine whether the locomotion effects are due to an alteration in neuromuscular transmission or in neuron to neuron transmission, or both.

### ACR-23 function is modulated by RIC-3

The assembly of many nAChRs requires the assistance from chaperones among which RIC-3 represents an example in *C. elegans* [6]. We found that a *ric-3* mutant is slightly resistant to monepantel suggesting that the RIC-3 protein may be required for the function of ACR-23. Unexpectedly, the growth of *ric-3; acr-23* double mutants is more robust in the presence of monepantel than in its absence. We propose that monepantel could activate other nAChR channels that are poorly expressed or weakly functional in a *ric-3* mutant background. However, the nAChR proteins DES-2, DEG-3, ACR-5 and ACR-18 appear not to be such candidates, since their corresponding mutant alleles do not hinder the increased growth rate on monepantel. Therefore, proteins providing more efficient growth in the presence of the anthelmintic remain to be discovered. Such proteins may represent additional targets of monepantel. Interestingly, we have noticed that in the absence of monepantel, *acr-5; ric-3; acr-23* triple mutants grow more efficiently than *ric-3; acr-23* double mutants. Therefore we hypothesize that ACR-5 might compete for a chaperone that is distinct from RIC-3 and which is required for efficient growth.

We have shown that the levels of ACR-23 are not significantly modulated by monepantel and by RIC-3. However, the weak resistance of *ric-3* mutants to monepantel indicates that RIC-3 functions as a minor player in the response to the anthelmintic. Nevertheless, it remains possible, that RIC-3 regulates the expression of ACR-23 but within a range that could not be detected in our assay. Finally, we also consider the possibility that proteins other than RIC-3 and ACR-23 could be required for the response to monepantel. Such proteins could be encoded by additional genes, which confer resistance to monepantel, but do not correspond to the *acr-23* open reading frame [2]. We predict that the cloning of such genes will uncover new targets of monepantel.
